# Mercurials in Dysentery

**Published:** 1852-10

**Authors:** 


*Mercurials in Dysentery.* — We insert with pleasure a second communication by Dr. Hunt, on the above subject, which will be found in the original department of this No. If our criticisms always succeed in provoking a repetition of the favors of correspondents who write as ably as does Dr. H., we shall have every reason to be satisfied with the results. Although we cannot concur with him in the hepatic pathology, we would commend his practical views to the candid consideration of our readers.

